# Role of mineral nutrition in alleviation of heat stress in cotton plants grown in glasshouse and field conditions

**DOI:** 10.1038/s41598-019-49404-6

**Published:** 2019-09-10

**Authors:** Muhammad Sarwar, Muhammad Farrukh Saleem, Najeeb Ullah, Shafaqat Ali, Muhammad Rizwan, Muhammad Rizwan Shahid, Mohammed Nasser Alyemeni, Saud A. Alamri, Parvaiz Ahmad

**Affiliations:** 1 Agronomic Research Institute, Ayub Agricultural Research Institute, Faisalabad, Pakistan; 2 Department of Agronomy, University of Agriculture Faisalabad, 38040 Faisalabad, Pakistan; 3 Queensland Alliance for Agriculture and Food Innovation | Centre for Plant Science, University of Queensland Wilsonton Heights, Toowoomba, QLD 4350 Australia; 4 Department of Environmental Sciences and Engineering, Government College University AllamaIqbal Road, 38000 Faisalabad, Pakistan; 5 Institute of Soil and Environmental Sciences, University of Agriculture, Faisalabad, 38000 Pakistan; 6 Department of Botany and Microbiology, College of Science, King Saud University, Riyadh, Saudi Arabia; 7 Department of Botany, S.P. College, Maulana Azad Road, Srinagar, Jammu and Kashmir 190001 India

**Keywords:** Abiotic, Heat

## Abstract

Coincidence of high temperature with terminal reproductive pheno-stages of cotton is chief constraint to achieve yield potential. This high temperature interfere plant defensive system, physiological process, water relations and lint yield production. In this study, we modulated the detrimental outcomes of heat stress on cotton through the foliar spray of nutrients. Cotton crop was exposed to sub-optimal and supra-optimal thermal regimes for a period of one week at squaring, flowering and boll formation stages under glass house and field conditions. Foliar spray of potassium (K-1.5%), zinc (Zn-0.2%) and boron (B-0.1%) were applied at three reproductive stages one day prior to expose high temperature regimes. High temperature increased lipid membrane damage through increased malondialdehyde (MDA) contents in cotton leaves. High temperature stress also reduced leaf chlorophyll contents, net photosynthetic rate, stomatal conductance, water potential, averaged boll weight (g) and seed cotton yield per plant. Various nutrients variably influenced growth and physiology of heat-stressed cotton plants. Zinc outclassed all other nutrients in increasing leaf SOD, CAT, POX, AsA, TPC activity, chlorophyll contents, net photosynthetic rate, stomatal conductance, water potential, boll weight and seed cotton yield per plant. For example, zinc improved seed cotton yield under supra-optimal thermal regime by 17% and under sub-optimal thermal regime by 12% of glasshouse study while 19% under high temperature sowing dates of field study than the water treated plants under the same temperatures. Conclusively, increasing intensities of temperature adversely affected the recorded responses of cotton and exogenous application of Zn efficaciously alleviated heat induced perturbations. Moreover, exogenous nutrients mediated upregulations in physiochemical attributes induced heat tolerance at morphological level.

## Introduction

Temperature is prophesied to rise by 5.8 °C till 2100 and 2.6 °C up to 2050 owing to global warming^1^. Heat waves along with more number of warm days and nights have been increased in most part of the world^2^. Cotton crop being native of semiarid regions is highly prone to confront with high temperature at the terminal reproductive stages^3^. Coincidence of high temperature with reproductive stages of cotton is a chief hindrance to accomplish yield potential in sub-continent. Since, temperature rises to 47 °C in May–June while accompanying high humidity in July–August develops a death-valley for cotton influencing all reproductive stages of cotton crop^4^. Heat stress mediated impairment in biosynthesis of antioxidants escalates the synthesis of reactive oxygen species (ROS) and thus induces oxidative stress^4,5^. Consequently, a cascade of reactions consequence into plethora of ROS that aggravate lipid peroxidation, excessive synthesis of malondialdehyde contents (MDA)^6^ and ultimately disruption in stability of membranes. Henceforth, the biosynthesis of chlorophyll contents decreases while degradation aggravates under heat triggered oxidative stress^7^. Whereas, impairment in biosynthesis of chlorophyll ultimately down regulates photosynthesis, translocation of assimilates to reproductive organs and accelerates senescence^8,9^. Moreover, heat stress also disrupts stomatal movement and ultimately consequence into poor gaseous exchange. Cotton leaves depict optimum stomatal conductance in temperature ranges 28–30.1 °C^10^ which starts to decline as temperature rises to 36 °C. Contrarily, as the temperature rises above 40 °C, stomata remain open and photosynthesis is impaired even if soil is well watered. Concurrently, high temperature stress mediated incongruities in stomatal movements disrupts the water relations resulting into reduction of growth^11,12^. Perturbations in biochemical attributes ultimately affect morphological attributes. The optimum temperature for development of boll ranges 25.5 °C–29.5 °C. While, boll weight is adversely affected as temperature rises above 25.5 °C–29.5 °C^13^. Similarly, each 1 °C rise of temperature above day maximum temperature decreases seed cotton yield by 110 kg ha^−1^ ^14^. Abiotic stresses along with oxidative stress and nutrient deficiency are the major causes of yield reduction throughout world^15^. Exogenous application of nutrients might prove a potent tool to alleviate deleterious impacts of heat^16^. Moreover, heat triggered decrease in uptake of nutrients from soil under heat stress further enhance the importance of exogenous supply of nutrients. Moreover, foliar applied nutrients produce higher yield and better-quality produce on alkaline calcareous soils^17^. Potassium and zinc are immobile within calcareous soils while B availability is also an issue in these soils^18–20^. Contrarily, potassium, zinc and boron are required in high quantity during the stress conditions. Potassium, zinc and boron modulates biochemical changes through antioxidant enzymes^21–23^ whereas, the exogenous application of potassium, zinc and boron up regulates the biosynthesis of chlorophyll which ultimately delays senescence enhances their quantity; consequently, improves the photosynthetic rate and the photosynthetic enzymes^24^. Likewise, potassium and zinc mediated regulations in water relations confer heat tolerance by sustaining water and osmotic potential of cell under stress conditions. While, boron availability enhances stomatal opening and thus regulate gaseous exchange under stressed environment^25,26^.

Therefore, considering the crucial role of macro (K) and micro nutrients (Zn and B) in protecting crops from extensive range of abiotic stresses, exogenous application under stress conditions might prove a potent tool to alleviate adverse impact of stress. The present study compares the potential role of foliar spray of macronutrient (potassium) in photosynthesis, regulation of water relations and stomatal conductance and micronutrients (zinc and boron) in reproduction and antioxidants. These physiochemical regulations might induce tolerance in morphological attributes of cotton crop exposed to different thermal regimes at squaring, flowering and boll formation. Hence, series of glasshouse and field experiments were conducted with objectives to (1) see the effect of different temperature regimes on leaf physiology and lint yield of cotton and to (2) reveal the role of macro and micro nutrients (K, Zn and B) for alleviation the impact of high temperature stress.

## Materials and Methods

### Glasshouse experiment

The glasshouse experiment was conducted at University of Agriculture Faisalabad (UAF), Pakistan. The experiment was performed during summer 2012. The seed of medium heat tolerant variety (AA-802) was collected from Ali Akbar Enterprises for this study. Soil properties and growth condition were same as have been reported in an earlier study^4^. Four seeds were sown at 2 cm depth had 12 hours pre-soaking in tap water. At four leaf stage of the seedlings, extra plants were thinned left only one plant in each pot. Treatments were comprised of optimal temperature (32/20 ± 2 °C day/night temperature or no stress), sub-optimal temperature (38/24 °C ± 2 °C or medium intensity heat stress) and supra-optimal temperature (45/30 °C ± 2 °C or high intensity heat stress); exogenously applied nutrients viz. water spray (control), foliar spray of K @ 1.5%, foliar spray of Zn @ 0.2% and foliar spray of B @ 0.1%. One day before shifting the pots to medium and high temperature chambers, the plants were sprayed with either of water (control), potassium (1.5%), zinc (0.2%) and boron (0.1%). Foliar concentrations of these nutrients were optimized in the preliminary glasshouse and field experiments (data not shown). All the plants were grown at 32/20 °C up to 30 DAS (before initiation of squaring). After that, pots were divided into 3 sets, each set was consisted of 20 pots which were transferred to growth chambers maintained for different temperature. First set of 20 pots was exposed to heat stress at squaring, 2^nd^ set at flowering and 3^rd^ set at boll formation. Heat stress was imposed for a period of one week at squaring, flowering and boll formation and data recorded were averaged across the three reproductive stages (squaring, flowering and boll formation). Samples from the youngest fully expanded leaves were collected immediately after removing the pots from stress, stored in liquid nitrogen and processed to record various attributes. The experiment was conducted using completely randomized design with split arrangement and replicated four times. Varying temperature regimes were imposed in main pots and nutrients were foliar applied in split pots.

### Field experiment

The field experiments were conducted at Agronomic Research Area, University of Agriculture Faisalabad, Pakistan during 2012 to 2013. The meteorological data were collected by the Meteorological Observatory of the Department of Agronomy, UAF. Treatments were comprised of sowing dates in main plots viz. early April (medium temperature at squaring, flowering and boll formation), early May (high temperature at squaring, flowering and boll formation) and mid-June (optimum temperature at squaring, flowering and boll formation). While, split plot treatments were consisted of foliar sprays of K, Zn and B viz. water spray, foliar spray of K @ 1.5%, foliar spray of Zn @ 0.2% and foliar spray of B @ 0.1%. Different sowing times were referred as thermal regimes. The varying temperature was recorded at squaring, flowering and boll formation stages. Three sowing times (April 2, May 3 and June 17 during 2012 and April 4, May 2 and June 19 during 2013) were selected based on previous five years’ climate data. Sowing of both experiments was done on sandy clay loam soil at times as per treatments during 2012 and 2013. Seed of cotton variety (AA- 802) was collected from Ali Akbar Enterprises during both years of study. Crop was planted with manual dibbling having 75 cm apart ridges and plant to plant distance was 30 cm. Weeds were controlled by two hoeing i.e. at squaring (35 days after planting) and at flowering (60 days after planting) while the sucking insects and boll worms were controlled with insecticides. Nine irrigations were applied as per crop requirement keeping in view the reproductive stages having heat stress at different sowing dates to avoid the drought stress during heat stress periods. The data of physiological parameters were recorded across the three reproductive stages of cotton i.e. squaring, flowering and boll formation. June thermal regime (late sown crop) was considered control, as it provided optimal temperature at all reproductive stages, while April (early sowing) and May sown crops were experienced sub and supra-optimal temperatures at three reproductive stages (Table 1). The experiment was laid out in randomized complete design with split treatment structure having three replications. Sowing dates were randomized in main and exogenous nutrients in split plots.

**Table 1:** Variable temperatures (thermal regimes) during one week of study under field conditions at three reproductive stages of cotton.

Growth phases	2012			2013		
	April	May	June	April	May	June
Squaring (initiation)	35.0–38.0 °C	38.5–45.0 °C	34.0–39.0 °C	33.0–39.0 °C	39.0–44 °C	34.0–37.9 °C
Flowering (initiation)	38.0–44.0 °C	43.5°–45.0 °C	33.0–38.0 °C	37.0–45.7 °C	39.0–41.3 °C	31.8–37.0 °C
Boll formation	38.0–41.0 °C	42.5°–46.0 °C	33.0–39.0 °C	33.9–44.9 °C	32.0–39.2 °C	35.5–37.2 °C

Optimal = 34–38 °C, sub optimal = 39–41 °C and supra-optimal up to 46 °C

Maximum temperature ranges of treatment period - one week. At squaring stage, temperature of the May sown crop was raised to supra optimal condition during both years of study. Flowering stage of April-2012, May-2012 and April-2013 sown crops experienced heat stress period, while sub optimal temperature prevailed at same stage of May-2013 sown crop. Heat stress periods were also observed at boll formation May-2012 and April-2013 sown crops while boll formation stage of April-2012 sown crop faced sub optimal conditions. June planted crop faced optimal temperatures at all reproductive stages during both years of study and thus considered optimal or control sowing date.

### Observations

#### Biochemical assays

Leaves samples weighing 0.5 g were extracted in with 10 ml of phosphate buffer (pH 7.8) for the extraction of enzymes. The supernatant was used for enzyme determination after centrifuge and the residues were discarded. The extracted material was stored at 4 °C^27^. The samples for all enzymatic and non-enzymatic antioxidants were pipetted into 96-well plates. The plates were, then, read by micro plate reader (ELX800, Bio-Tek Instruments, Inc., Winooski, VT, USA) at different wavelengths. Superoxide dismutase contents were determined by^28^ method. Superoxide dismutase was quantified as enzymes units that inhibited photo reduction of nitrobluetetrazolium (NBT) and recorded the absorbance at 470 nm. While, CAT was measured as enzymes units that converted H_2_O_2_ to H_2_O and O_2_ using the protocol as described by Liu^29^. The reaction mixture [50 mM phosphate buffer (pH 7) + 5.9 mM H_2_O_2_] was mixed with 0.1 mL enzyme extract and read the absorbance at 240 nm. Peroxidase contents were determined using method as given by^29^. Peroxidase was quantified as units of enzymes that oxidized guaiacol. The reaction mixture was comprised of 50 mM phosphate buffer (pH 5) + 40 mM H_2_O_2_ + 20 mM guaiacol and 0.1 mL of enzyme extract per each sample. The absorbance was recorded at wavelength of 470 nm.

For the estimation of ascorbic acid, 900 µL dist. H_2_O + 100 µL sample extract + 1 mL dichlorophenol-indophenol + 100 µL 0.1% Meta H_3_PO_4_ were mixed in a test tube and absorbance was recorded at 520 nm^30^. Folin-Ciocalteu (FC) reagent method was used for the determination of TPC^31^. Leaves samples of 0.5 g weight were extracts with 80% acetone (10 mL) and centrifuged. Enzyme extract (20 µL) + FC-reagent (100 µL) + 1.5 mL water were mixed in a cuvette and placed for 30 minutes. Then, added 700 mM Na_2_CO_3_ and incubated at room temperature for period of 2 hours. The absorbance was taken at 765 nm having 200 µL sample in each well. MDA contents in cotton leaves were determined according following the procedure as adapted by^32^. Leaf sample (0.5 g) was homogenized in 10 ml of 0.1% trichloroacetic acid (TCA) solution and centrifuged at 12000 × g for 15 minutes. For each mL of extract 4.5 ml of thiobarbituric acid (0.5%) was used with the reaction mixture and heated at 95 °C for 30 min and cooled. The absorbance was taken at 532 and 600 nm and MDA concentration was determine using formula:

$${\rm{MDA}}\,{\rm{level}}\,({\rm{nmol}})=\frac{\Delta ({\rm{A}}532\,{\rm{nm}}-{\rm{A}}600\,{\rm{nm}})}{156\times {10}^{5}}$$

A = Absorption coefficient with the value of 156 mm^−1^ cm^−1^.

### Chlorophyll contents

Cotton leaves (0.5 g) were ground in 10 ml of 80% cold acetone and the tubes were stored in dark at 20 °C overnight, indicating minor modifications of previously described method^33^. The mixture was filtered through a Whatman No 1. A blank with 80% acetone was run; the measurements were taken at 645 and 663 nm through a spectrophotometer. The chlorophyll contents were calculated from the formula:

$$Chl\,a\,(\frac{mg}{g}FW)=[12.7\,(OD\,663)-2.69\,(OD\,645)]\,\ast \,\frac{V}{1000}\,\ast \,W$$

$$Chl\,b\,(\frac{mg}{g}FW)=[22.9\,(OD\,645)-4.68\,(OD\,663)]\,\ast \,\frac{V}{1000}\,\ast \,W$$

where W is the weight of leaf sample while V is the volume of sample used in spectrophotometer (U-2001, Hitachi, Japan).

### Net photosynthetic rate and stomatal conductance

Net photosynthetic rate and stomatal conductance was determined at three reproductive stages of cotton crop through a portable infrared gas analyzer (LCiAnalyser having Broad Head, Part Number LCi-002/B with Serial Number 32455). The Pn was measured at each reproductive stage after 3 days of spray between 10:00 a.m. to 12:00 p.m. on fully expanded young leaves.

### Water relations

Leaf samples (Leaf water and osmotic potential) were collected at pre-dawn (6:00 h) as previously described^34^. Leaf water potential was determined through Scholander type pressure chamber (ARIMAD 2, Korea) following methodology as described by^35^ instantly after sampling. While, leaves were stored at −20 °C for a period of one week, then thawed, extracted sap and determined the osmotic potential with the help of osmometer (Osmomat 030).

### Agronomic attributes

Ten plants were randomly selected in each experimental unit of filed study while five plants were selected from five random pots of optimal, sub and supra-optimal thermal regimes of glass house study. Averaged boll weight was noted by dividing total seed cotton yield per plant with total number of bolls. While, seed cotton yield was weighed separately for each plot/pot and converted to per hectare yield from each plot.

### Statistical analysis

Analysis of variance was employed to determine significance (F-test) of heat and foliar nutrients. While, means of treatments were compared using least significant difference test (p ≤ 0.05). Correlation among the varying response variables was computed using means of treatments calculated across the three blocks. Strength, type and significance of correlation was determined using STATISTIX 8.1 software (Analytical Software, Tallahassee, Florida, USA). Number of pairs of observations (n) to determine correlation were 36 (replications × main plots × sub plots). Figures were developed using MS excel-2016.

## Results

### Green house experiment

**Figure 1:**
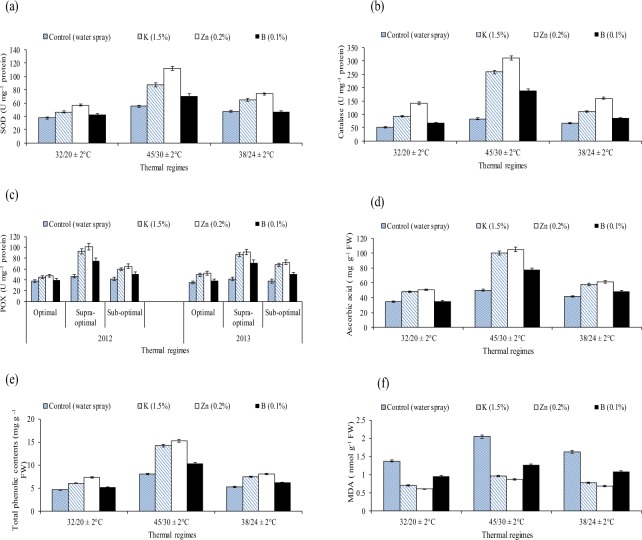
Effect of different thermal regimes and nutrients’ spray on superoxide dismutase (SOD), catalase (CAT), peroxidase (POX U mg^−1^ protein), ascorbic acid (AsA mg g^−1^ FW), total phenolic contents (TPC mg g^−1^ FW) and malondialdehyde contents (MDA nmol g^−1^ FW), (averaged across of squaring, flowering and boll formation stages) of cotton leaves under glass house conditions.

**Figure 2:**
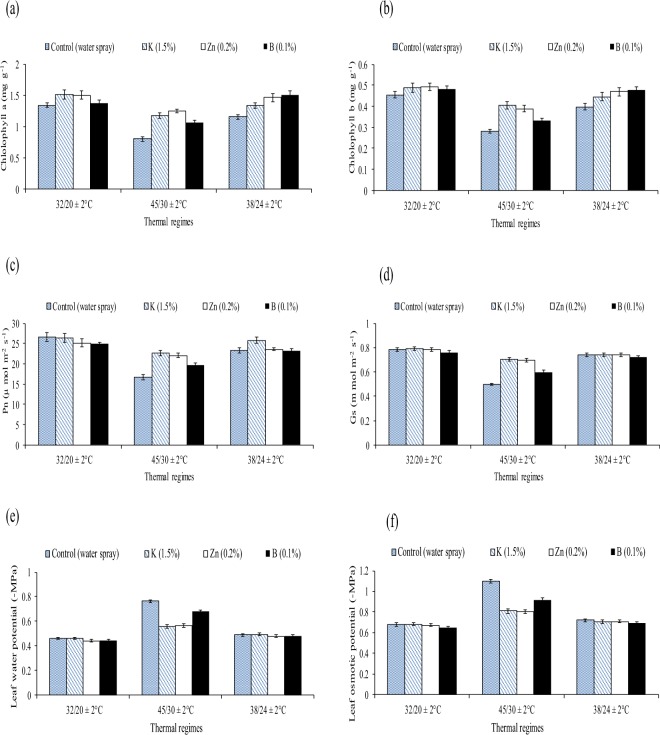
Effect of different thermal regimes and nutrients’ spray on chlorophyll contents (a + b) (mg g^−1^FW), net photosynthetic rate-Pn (µmol m^−2^ sec^−1^), FW), stomatal conductance (Gs m mol m^−2^ s^−1^), leaf water potential (−MPa) and leaf osmotic potential (−MPa) (averaged across of squaring, flowering and boll formation stages) of cotton leaves under glass house conditions.

**Figure 3:**
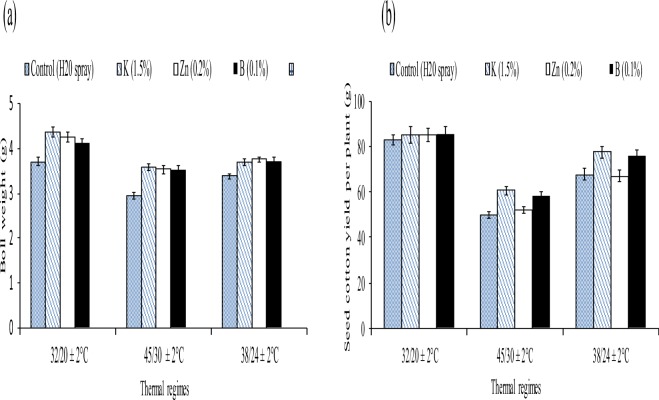
Effect of different thermal regimes and nutrients’ spray on averaged boll weight (g) and seed cotton yield per plant (g) of cotton crop under glass house conditions.

Significant interaction of heat and foliar nutrients was recorded for all the studied attributes. Supra optimal regime followed by sub optimal regime triggered increase in antioxidants, MDA and decrease in chlorophyll contents, photosynthetic rate, gaseous exchange components, water relations, boll weight and seed cotton yield over the optimal temperature regime. Foliar applied ‘0.2% Zn’ depicted outstanding results regarding the alleviation of adverse impacts of heat, followed by ‘1.5% K’ and ‘0.1% B’ for all the studied attributes (Tables 2–4, Figs 1–3).

**Table 2:** Effect of different thermal regimes and nutrients’ spray on superoxide dismutase (SOD), catalase (CAT), peroxidase (POX U mg^−1^ protein), ascorbic acid (AsA mg g^−1^ FW), total phenolic contents (TPC mg g^−1^ FW) and malondialdehyde contents (MDA nmol g^−1^ FW), (averaged across of squaring, flowering and boll formation stages) of cotton leaves under glass house conditions.

Thermal regimes	Nutrients	SOD	CAT	POX	ASA	TPC	MDA
32/20 °C	Control	37.71 d ± 2.0	51.33 d ± 3.13	34.89 c ± 0.52	137.92 d ± 8.63	4.63 d ± 0.52	1.37 a ± 0.65
	Potassium (1.5%)	46.31 b ± 2.5	91.85 b ± 5.97	47.96 b ± 0.74	195.58 b ± 10.25	6.06 b ± 0.30	0.70 c ± 0.40
	Zinc (0.2%)	56.71 a ± 3.0	140.39 a ± 8.82	50.96 a ± 0.98	207.39 a ± 12.35	7.27 a ± 0.41	0.60 d ± 0.35
	Boron (0.1%)	42.78 bc ± 2.3	68.57 c ± 4.35	35.23 c ± 0.56	177.94 c ± 8.75	5.18 c ± 0.25	0.96 b ± 0.48
45/30 °C	Control	55.16 d ± 2.8	83.10 d ± 4.54	50.17 d ± 0.78	170.12 c ± 11.20	8.00 c ± 0.41	2.04 a ± 0.14
	Potassium (1.5%)	87.41 b ± 5.1	258.73 b ± 13.65	100.62 b ± 1.5	328.44 a ± 22.10	14.25 b ± 0.70	0.96 c ± 0.051
	Zinc (0.2%)	111.83 a ± 6.2	310.77 a ± 16.31	104.79 a ± 1.3	337.90 a ± 20.30	15.23 a ± 0.84	0.87 d ± 0.044
	Boron (0.1%)	70.66 c ± 3.7	189.87 c ± 10.19	77.48 c ± 0.92	250.51 b ± 14.50	10.38 d ± 0.52	1.26 b ± 0.065
38/24 °C	Control	47.23 d ± 2.4	66.17 d ± 3.60	41.70 d ± 0.58	156.32 c ± 8.4	5.24 d ± 0.32	1.63 a ± 0.70
	Potassium (1.5%)	64.70 b ± 3.5	109.91 b ± 6.63	57.99 b ± 0.71	228.12 a ± 14.45	7.48 b ± 0.40	0.77 c ± 0.50
	Zinc (0.2%)	73.58 a ± 3.8	160.17 a ± 9.22	61.25 a ± 0.88	230.39 a ± 16.76	8.06 a ± 0.50	0.69 d ± 0.41
	Boron (0.1%)	51.29 c ± 2.8	85.16 c ± 4.62	48.71 c ± 0.64	205.57 b ± 11.20	6.21 c ± 0.35	1.08 b ± 0.64
	LSD	3.65	5.72	2.94	11.78	0.49	0.068

Values are the means of three replications (n = 4) ± SE and variants possessing the same letters are not statistically significant at *P* < *0.05*. Main factors and interaction are significant at *P* < *0.01*. Lettering is done separately for each thermal regime using the LSD of the interaction between thermal regimes and nutrients’ spray.

**Table 3:** Effect of different thermal regimes and nutrients’ spray on chlorophyll contents (a + b) (mg g^−1^FW), net photosynthetic rate-Pn (µmol m^−2^ sec^−1^), FW), stomatal conductance (Gs m mol m^−2^ s^−1^), leaf water potential (−MPa) and leaf osmotic potential (−MPa) (averaged across of squaring, flowering and boll formation stages) of cotton leaves under glass house conditions.

Thermal regimes	Nutrients	Chlorophyll a	Chlorophyll b	Pn	Gs	Water Potential	Osmotic potential
32/20 °C	Control	1.34 b ± 0.070	0.46 ab ± 0.27	26.69 a ± 0.63	0.79 a ± 0.042	0.46 a ± 0.041	0.68 a ± 0.034
	Potassium (1.5%)	1.51 a ± 0.073	0.48 a ± 0.30	26.38 a ± 0.67	0.79 a ± 0.040	0.46 a ± 0.040	0.68 a ± 0.032
	Zinc (0.2%)	1.50 a ± 0.076	0.49 a ± 0.32	26.20 a ± 0.57	0.78 a ± 0.035	0.44 a ± 0.039	0.67 a ± 0.035
	Boron (0.1%)	1.37 b ± 0.068	0.48 a ± 0.26	26.04 a ± 0.12	0.77 a ± 0.036	0.44 a ± 0.038	0.66 a ± 0.031
45/30 °C	Control	0.80 c ± 0.051	0.28 c ± 0.20	16.74 c ± 0.37	0.50 c ± 0.024	0.76 a ± 0.070	1.10 a ± 0.52
	Potassium (1.5%)	1.18 a ± 0.062	0.41 a ± 0.23	22.66 a ± 0.40	0.71 a ± 0.037	0.56 c ± 0.052	0.81 c ± 0.040
	Zinc (0.2%)	1.25 a ± 0.067	0.39 a ± 0.21	22.11 a ± 0.37	0.70 a ± 0.033	0.57 c ± 0.055	0.80 c ± 0.038
	Boron (0.1%)	1.07 b ± 0.055	0.33 b ± 0.19	19.79 b ± 0.29	0.60 b ± 0.031	0.68 b ± 0.064	0.91 b ± 0.045
38/24 °C	Control	1.16 c ± 0.059	0.40 c ± 0.22	22.29 b ± 0.35	0.74 a ± 0.034	0.49 a ± 0.044	0.72 a ± 0.030
	Potassium (1.5%)	1.33 b ± 0.063	0.45 ab ± 0.25	25.55 a ± 0.49	0.74 a ± 0.037	0.49 a ± 0.045	0.71 a ± 0.034
	Zinc (0.2%)	1.46 a ± 0.079	0.47 a ± 0.28	25.33 a ± 0.16	0.74 a ± 0.038	0.48 a ± 0.043	0.71 a ± 0.035
	Boron (0.1%)	1.50 a ± 0.086	0.48 a ± 0.31	23.87 b ± 0.37	0.72 a ± 0.033	0.48 a ± 0.046	0.70 a ± 0.035
	LSD	0.080	0.023	1.38	0.032	0.021	0.031

Values are the means of three replications (n = 4) ± SE and variants possessing the same letters are not statistically significant at *P* < *0.05*. Main factors and interaction are significant at *P* < *0.01*. Lettering is done separately for each thermal regime using the LSD of the interaction between thermal regimes and nutrients’ spray.

**Table 4:** Effect of different thermal regimes and nutrients’ spray on averaged boll weight (g) and seed cotton yield per plant (g) of cotton crop under glass house conditions.

Thermal regimes	Nutrients	Boll Weight (g)	SCY
32/20 °C	Control	3.70 c ± 0.15	83.22 a ± 3.1
	Potassium (1.5%)	4.36 a ± 0.22	84.99 a ± 2.9
	Zinc (0.2%)	4.25 a ± 0.20	85.05 a ± 4.1
	Boron (0.1%)	4.13 ab ± 0.18	85.47 a ± 4.2
45/30 °C	Control	2.96 b ± 0.14	49.99 c ± 2.7
	Potassium (1.5%)	3.59 a ± 0.22	60.80 a ± 3.3
	Zinc (0.2%)	3.54 a ± 0.16	58.17 a ± 2.8
	Boron (0.1%)	3.54 a ± 0.17	53.67 b ± 2.7
38/24 °C	Control	3.38 b ± 0.11	67.69 c ± 4.4
	Potassium (1.5%)	3.70 a ± 0.19	77.45 a ± 4.0
	Zinc (0.2%)	3.75 a ± 0.15	75.92 a ± 3.8
	Boron (0.1%)	3.73 a ± 0.14	71.34 b ± 3.7
	LSD	0.20	2.99

Values are the means of three replications (n = 4) ± SE and variants possessing the same letters are not statistically significant at *P* < *0.05*. Main factors and interaction are significant at *P* < *0.01*. Lettering is done separately for each thermal regime using the LSD of the interaction between thermal

Superoxide dismutase contents were improved by 46% and 25% while catalase contents were increased by 61% and 29% when compared the controls of supra and sub-optimal thermal regimes with the control of optimal thermal regime averaged across of three reproductive stages. Similarly, POX, AsA and TPC contents were increased under sub and supra-optimal thermal regimes. Biosynthesis of SOD was enhanced by 32% and 56% with ‘0.2% Zn’ compared to water spray under sub and supra-optimal thermal regimes. Similarly, ‘0.2% Zn’ instigated improvements in biosynthesis of CAT by 60% and 73% under sub and supra-optimal temperature regime than their respective water treated plants. The SOD and CAT contents were also increased under optimal thermal regime but the effect was more pronounced under sub and supra-optimal thermal regimes. Whereas, POX was up-regulated by 31, 32 and 52% under optimal, sub optimal and supra optimal temperature regimes, respectively with ‘0.2% Zn’ compared to water spray. Likewise, Zn also enhanced the POX, AsA and TPC contents under optimal, sub optimal and supra optimal temperature regimes. Moreover, MDA synthesis was downregulated by 56%, 58% and 57% with ‘0.2% Zn’ compared to ‘water treated plants’ under optimal, sub optimal and supra optimal temperature regimes, respectively (Table 2, Fig. 1).

The Chlorophyll *a* and *b* contents were reduced by 15% and 66% under the controls (water treated plants) of sub and supra-optimal thermal regimes when compared with water treated plants of optimal thermal regime. Net photosynthetic rate was reduced by 20% and 60% when compared the water treated plants of sub and supra-optimal thermal regimes with water treated plants of optimal thermal regime (averaged across of three reproductive stages). Similarly, stomatal conductance and water potential were reduced while osmotic potential was increased under sub and supra optimal thermal regimes.

The comparative improvements in chlorophyll *a*, *b* contents, Pn and Gs owing to ‘0.2% Zn’ with respect to water spray were statistically higher under sub and supra optimal thermal regimes. For example, Zn improved Chlorophyll *a* content by 23% and 46% under sub and supra optimal thermal regimes than water treated plants. Similarly, zinc also improved chlorophyll *b* contents, Pn, Gs and water potential under sub and supra optimal thermal regimes. (Table 3, Fig. 2).

Although, the seed cotton yield (SCY) was reduced by 66% and 23% in the controls of supra and sub-optimal thermal regimes than the control of optimal thermal regime. The similar reduction was found for averaged boll weight. The foliar spray of three nutrients (K, Zn and B) improved SCY by 21%, 16% and 7% in the high temperature regime than water treated plants. Likewise, the nutrients improved the averaged boll weight under high temperature regime (Table 4, Fig. 3).

### Field experiment

**Figure 4:**
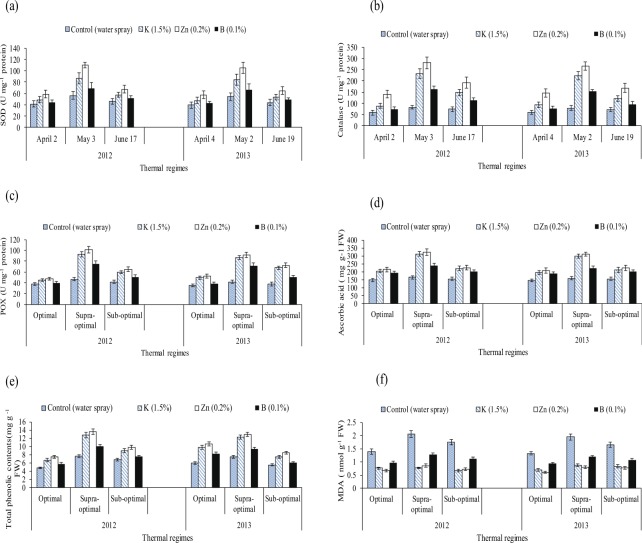
Effect of different thermal regimes and nutrients’ spray on effect of different thermal regimes and nutrients’ spray on superoxide dismutase (SOD), catalase (CAT), peroxidase (POX U mg^−1^ protein), ascorbic acid (AsA mg g^−1^ FW), total phenolic contents (TPC mg g^−1^ FW) and malondialdehyde contents (MDA nmol g^−1^ FW), (averaged across of squaring, flowering and boll formation stages) of cotton leaves under field conditions during 2012 and 2013.

**Figure 5:**
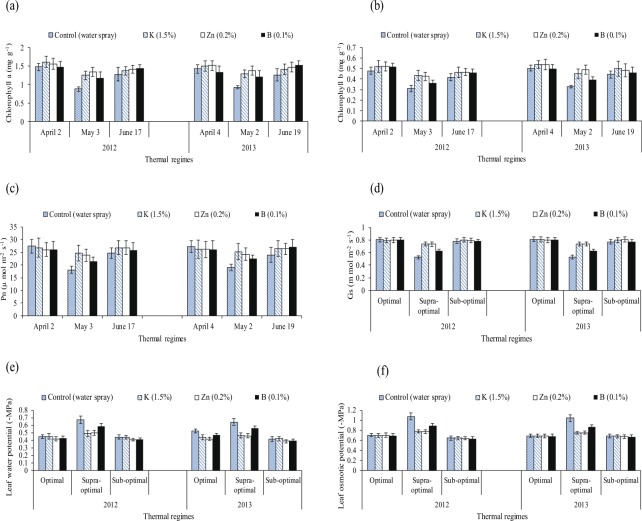
Effect of different thermal regimes and nutrients’ spray on chlorophyll contents (a + b) (mg g^−1^FW), net photosynthetic rate-Pn (µmol m^−2^ sec^−1^), FW), stomatal conductance (Gs m mol m^−2^ s^−1^), leaf water potential (−MPa) and leaf osmotic potential (−MPa) (averaged across of squaring, flowering and boll formation stages) of cotton leaves during 2012 and 2013.

**Figure 6:**
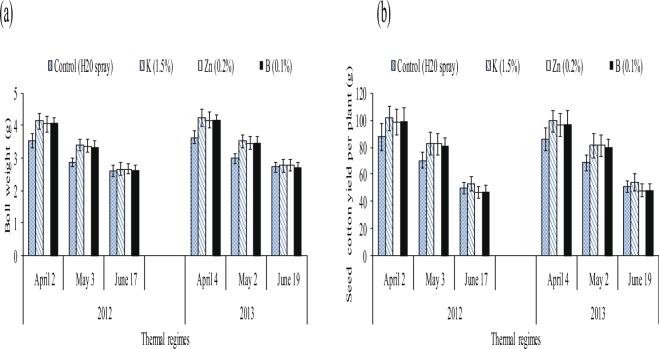
Effect of different thermal regimes and nutrients’ spray on averaged boll weight (g) and seed cotton yield per plant (g) of cotton crop under field conditions during 2012 and 2013.

Supra optimal temperature regimes were relatively more detrimental, and it was followed by sub optimal temperature regimes (Table 1). While, exogenously applied nutrients depicted significant improvements compared to water spray (control). However, relatively more promising results were obtained with ‘0.2% Zn’, followed by ‘1.5% K’, ‘0.1% B’ and water spray. The recorded improvements by the application of exogenous nutrients differ significantly under varying temperature regimes. (Tables 5–8, Figs 4–6).

**Table 5:** Effect of different thermal regimes and nutrients’ spray on superoxide dismutase (SOD), catalase (CAT), peroxidase (POX U mg^−1^ protein) and ascorbic acid (AsA mg g^−1^ FW) contents (averaged across of squaring, flowering and boll formation stages) of cotton leaves under field conditions during 2012 and 2013.

Thermal regimes	Nutrients	SOD 2012	SOD 2013	CAT 2012	CAT 2013	POX 2012	POX 2013	AsA 2012	AsA 2013
Optimal regimes of sowing dates	Control	41.33 cd ± 3.8	39.42 bc ± 3.5	58.11 cd ± 5.60	59.49 cd ± 5.60	37.35 ab ± 3.6	35.30 b ± 3.0	147.94 b ± 13.40	145.45 b ± 14.0
	Potassium (1.5%)	47.55 b ± 4.4	47.69 a ± 4.3	88.01 b ± 7.08	93.48 b ± 7.08	45.04 a ± 3.9	49.49 a ± 4.6	204.21 a ± 18.70	196.40 a ± 17.0
	Zinc (0.2%)	58.73 a ± 5.3	57.35 a ± 5.1	140.86 a ± 10.70	144.94 a ± 10.70	47.62 a ± 4.1	52.47 a ± 4.5	215.12 a ± 19.40	206.47 a ± 19.6
	Boron (0.1%)	44.10 bc ± 4.1	43.12 ab ± 3.7	73.11 bc ± 5.60	76.42 bc ± 5.60	39.67 a ± 3.7	38.07 b ± 3.6	192.34 a ± 17.40	187.12 a ± 18.20
Supra-optimal of sowing dates	Control	56.04 d ± 4.9	54.04 d ± 4.9	81.81 d ± 5.90	78.22 d ± 5.90	46.86 c ± 4.0	41.85 c ± 4.0	164.57 c ± 15.8	158.87 c ± 14.50
	Potassium (1.5%)	86.66 b ± 7.8	84.34 b ± 8.1	230.87 b ± 10.30	221.95 b ± 10.30	93.14 a ± 8.3	86.44 a ± 7.6	312.83 a ± 30.20	298.17 a ± 27.60
	Zinc (0.2%)	110.28 a ± 10.1	105.12 a ± 9.9	280.19 a ± 10.66	264.68 a ± 10.66	101.56 a ± 9.7	91.34 a ± 8.2	325.01 a ± 31.50	310.61 a ± 30.14
	Boron (0.1%)	69.17 c ± 6.3	66.75 c ± 6.1	161.45 c ± 4.07	153.64 c ± 4.07	74.61 b ± 6.6	71.32 b ± 6.5	239.03 b ± 22.60	221.04 b ± 20.90
Sub-optimal of sowing dates	Control	46.21 cd ± 4.3	43.79 cd ± 3.6	73.17 d ± 5.47	70.24 cd ± 5.47	41.89 bc ± 4.0	38.08 c ± 3.2	154.05 b ± 14.80	155.11 b ± 13.50
	Potassium (1.5%)	56.54 b ± 5.1	52.63 b ± 4.9	148.08 b ± 6.53	122.20 b ± 6.53	62.00 a ± 5.2	67.92 a ± 5.4	221.52 a ± 21.40	211.13 a ± 21.30
	Zinc (0.2%)	67.04 a ± 6.5	64.42 a ± 6.2	192.29 a ± 11.47	168.10 a ± 11.47	65.23 a ± 5.8	72.38 a ± 6.5	225.64 a ± 21.0	223.88 a ± 20.80
	Boron (0.1%)	51.56 bc ± 4.5	49.21 bc ± 4.8	112.44 c ± 7.17	95.41 c ± 7.17	50.43 b ± 4.6	51.00 b ± 4.2	201.08 a ± 18.40	200.43 a ± 17.80
	LSD	10.46	11.04	27.83	25.86	11.00	7.80	37.93	33.19

Values are the means of three replications (n = 3) ± SE and variants possessing the same letters are not statistically significant at *P* < *0.05*. Lettering is done separately for each thermal regime using the LSD of the interaction between thermal regimes and nutrients’ spray.

**Table 6:** Effect of different thermal regimes and nutrients’ spray on total phenolic contents (TPC mg g^−1^ FW), malondialdehyde contents (MDA nmol g^−1^ FW) and chlorophyll contents (a + b) (mg g^−1^FW) (averaged across of squaring, flowering and boll formation stages) of cotton leaves under field conditions during 2012 and 2013.

Thermal regimes	Nutrients	TPC 2012	TPC 2013	MDA 2012	MDA 2013	Chlorophyll a 2012	Chlorophyll a 2013	Chlorophyll b 2012	Chlorophyll b 2013
Optimal regimes of sowing dates	Control	4.86 b ± 0.37	5.98 c ± 0.45	1.39 a ± 0.87	1.32 a ± 0.97	1.48 ab ± 0.11	1.42 a ± 0.12	0.48 ab ± 0.39	0.50 a ± 0.42
	Potassium (1.5%)	6.40 a ± 0.52	9.82 a ± 0.80	0.76 c ± 0.065	0.70 c ± 0.62	1.62 a ± 0.13	1.50 a ± 0.11	0.52 a ± 0.45	0.54 a ± 0.50
	Zinc (0.2%)	7.40 a ± 0.60	10.60 a ± 0.91	0.66 c ± 0.052	0.60 c ± 0.48	1.56 a ± 0.10	1.52 a ± 0.13	0.53 a ± 0.47	0.54 a ± 0.48
	Boron (0.1%)	5.81 b ± 0.48	8.13 b ± 0.72	0.97 b ± 0.078	0.93 b ± 0.84	1.47 ab ± 0.12	1.38 a ± 0.09	0.52 a ± 0.50	0.50 a ± 0.35
Supra-optimal of sowing dates	Control	7.65 c ± 0.55	7.43 c ± 0.60	2.06 a ± 0.60	1.94 a ± 0.17	0.88 c ± 0.072	0.92 c ± 0.07	0.31 c ± 0.26	0.33 c ± 0.27
	Potassium (1.5%)	12.82 a ± 1.0	12.23 a ± 0.99	0.76 c ± 0.06	0.88 c ± 0.74	1.25 a ± 0.10	1.30 a ± 0.11	0.43 a ± 0.40	0.45 a ± 0.32
	Zinc (0.2%)	13.60 a ± 1.1	12.95 a ± 1.1	0.86 c ± 0.074	0.80 c ± 0.70	1.34 a ± 0.12	1.37 a ± 0.10	0.43 a ± 0.38	0.49 a ± 0.30
	Boron (0.1%)	9.97 b ± 0.80	9.29 b ± 0.80	1.28 b ± 0.94	1.19 b ± 0.10	1.18 ab ± 0.091	1.21 ab ± 0.08	0.36 b ± 0.30	0.39 b ± 0.25
Sub-optimal of sowing dates	Control	6.89 c ± 0.56	5.55 c ± 0.49	1.74 a ± 1.30	1.65 a ± 0.49	1.28 ab ± 0.11	1.26 b ± 0.09	0.42 bc ± 0.41	0.44 ab ± 0.36
	Potassium (1.5%)	8.93 a ± 0.75	7.53 a ± 0.62	0.67 c ± 0.62	0.83 c ± 0.77	1.37 a ± 0.10	1.41 a ± 0.11	0.47 a ± 0.45	0.50 a ± 0.39
	Zinc (0.2%)	9.80 a ± 0.85	8.42 a ± 0.72	0.72 c ± 0.68	0.76 c ± 0.66	1.41 a ± 0.13	1.47 a ± 0.13	0.47 a ± 0.44	0.48 a ± 0.41
	Boron (0.1%)	7.50 b ± 0.68	6.05 b ± 0.55	1.13 b ± 0.93	1.07 b ± 0.10	1.44 a ± 0.12	1.53 a ± 0.14	0.46 ab ± 0.30	0.48 a ± 0.38
	LSD	1.37	0.88	0.18	0.11	0.13	0.16	0.040	0.050

Values are the means of three replications (n = 3) ± SE and variants possessing the same letters are not statistically significant at *P* ˂ *0.05*. Lettering is done separately for each thermal regime using the LSD of the interaction between thermal regimes and nutrients’ spray.

**Table 7:** Effect of different thermal regimes and nutrients’ spray on net photosynthetic rate-Pn (µmol m^−2^ sec^−1^), FW), stomatal conductance (Gs m mol m^−2^ s^−1^), leaf water potential (−MPa) and leaf osmotic potential (−MPa) (averaged across of squaring, flowering and boll formation stages) of cotton leaves under field conditions during 2012 and 2013.

Thermal regimes	Nutrients	Pn 2012	Pn 2013	Gs 2012	Gs 2013	Water Potential 2012	Water Potential 2013	Osmotic Potential 2012	Osmotic Potential 2013
Optimal regimes of sowing dates	Control	27.43 a ± 2.4	27.27 a ± 2.5	0.81 a ± 0.076	0.81 a ± 0.075	0.45 a ± 0.039	0.46 a ± 0.041	0.70 a ± 0.065	0.69 a ± 0.062
	Potassium (1.5%)	26.83 a ± 2.1	26.18 a ± 2.2	0.79 a ± 0.073	0.81 a ± 0.077	0.45 a ± 0.035	0.44 a ± 0.038	0.70 a ± 0.062	0.69 a ± 0.065
	Zinc (0.2%)	26.05 a ± 2.3	26.07 a ± 1.9	0.80 a ± 0.071	0.80 a ± 0.075	0.42 a ± 0.040	0.42 a ± 0.036	0.70 a ± 0.069	0.68 a ± 0.059
	Boron (0.1%)	26.01 a ± 2.0	26.20 a ± 2.4	0.80 a ± 0.077	0.80 a ± 0.074	0.43 a ± 0.038	0.47 a ± 0.043	0.69 a ± 0.060	0.68 a ± 0.061
Supra-optimal of sowing dates	Control	18.06 c ± 1.6	18.92 c ± 1.3	0.52 c ± 0.049	0.53 c ± 0.049	0.67 a ± 0.057	0.64 a ± 0.059	1.07 a ± 0.091	1.04 a ± 0.091
	Potassium (1.5%)	24.62 a ± 1.9	25.20 a ± 1.8	0.73 a ± 0.068	0.74 a ± 0.069	0.49 c ± 0.042	0.46 c ± 0.044	0.78 c ± 0.072	0.75 c ± 0.070
	Zinc (0.2%)	23.76 a ± 1.8	24.26 a ± 2.1	0.73 a ± 0.069	0.73 a ± 0.071	0.50 c ± 0.046	0.46 c ± 0.041	0.78 c ± 0.068	0.75 c ± 0.068
	Boron (0.1%)	21.56 ab ± 1.6	22.10 ab ± 1.9	0.63 b ± 0.057	0.63 b ± 0.058	0.59 b ± 0.053	0.55 b ± 0.051	0.88 b ± 0.082	0.86 b ± 0.081
Sub-optimal of sowing dates	Control	24.58 ab ± 2.2	23.99 ab ± 2.2	0.78 a ± 0.073	0.77 a ± 0.071	0.44 a ± 0.038	0.42 a ± 0.037	0.65 a ± 0.057	0.68 a ± 0.061
	Potassium (1.5%)	26.83 a ± 2.5	26.56 a ± 2.5	0.80 a ± 0.076	0.80 a ± 0.075	0.44 a ± 0.041	0.42 a ± 0.038	0.64 a ± 0.059	0.68 a ± 0.058
	Zinc (0.2%)	27.80 a ± 2.6	26.41 a ± 2.3	0.79 a ± 0.074	0.81 a ± 0.079	0.41 a ± 0.038	0.39 a ± 0.033	0.64 a ± 0.062	0.68 a ± 0.063
	Boron (0.1%)	26.92 a ± 2.2	27.19 a ± 2.6	0.78 a ± 0.070	0.77 a ± 0.071	0.41 a ± 0.037	0.39 a ± 0.035	0.63 a ± 0.059	0.67 a ± 0.061
	LSD	2.98	3.01	0.096	0.089	0.08	0.073	0.10	0.11

Values are the means of three replications (n = 3) ± SE and variants possessing the same letters are not statistically significant at *P* < *0.05*. Lettering is done separately for each thermal regime using the LSD of the interaction between thermal regimes and nutrients’ spray.

**Table 8:** Effect of different thermal regimes and nutrients’ spray on averaged boll weight (g) and seed cotton yield per plant (g) of cotton crop under field conditions during 2012 and 2013.

Thermal regimes	Nutrients	Boll weight (g) 2012	Boll weight (g) 2013	SCY 2012	SCY 2013
April (High temperature) sub optimal	Control	3.53 a ± 0.31	3.64 b ± 0.32	87.60 b ± 8.4	85.60 b ± 8.1
	Potassium (1.5%)	4.13 a ± 0.38	4.24 a ± 0.39	101.34 a ± 9.9	99.34 a ± 9.4
	Zinc (0.2%)	4.05 a ± 0.40	4.16 a ± 0.37	98.44 a ± 9.2	97.62 a ± 9.2
	Boron (0.1%)	4.07 a ± 0.35	4.18 a ± 0.41	99.41 a ± 9.5	97.41 a ± 9.6
May (High temperature) supra	Control	2.88 b ± 0.23	2.99 b ± 0.26	70.11 b ± 6.3	68.45 b ± 6.4
	Potassium (1.5%)	3.41 a ± 0.29	3.52 a ± 0.32	82.89 a ± 7.7	81.22 a ± 7.8
	Zinc (0.2%)	3.36 a ± 0.31	3.46 a ± 0.29	82.37 a ± 7.9	81.37 a ± 7.5
	Boron (0.1%)	3.35 a ± 0.30	3.45 a ± 0.27	80.92 a ± 7.5	79.92 a ± 7.1
June (late sown as optimal)	Control	2.61 a ± 0.20	2.72 a ± 0.21	49.99 a ± 4.3	51.19 a ± 4.7
	Potassium (1.5%)	2.66 a ± 0.24	2.78 a ± 0.25	52.99 a ± 4.7	54.19 a ± 4.9
	Zinc (0.2%)	2.67 a ± 0.22	2.79 a ± 0.24	47.07 a ± 4.1	48.27 a ± 4.5
	Boron (0.1%)	2.61 a ± 0.18	2.73 a ± 0.26	47.33 a ± 4.0	48.53 a ± 4.3
	LSD	0.37	0.40	9.66	9.78

Values are the means of three replications (n = 3) ± SE and variants possessing the same letters are not statistically significant at *P* < *0.05*. Lettering is done separately for each thermal regime using the LSD of the interaction between thermal regimes and nutrients’ spray.

In the controls of supra and sub optimal thermal regimes of April and May sown crops, the SOD and CAT contents were increased by 37%, 36% and 22%, 11% (averaged across both years of study and of three developmental stages) than the water treated plants of optimal thermal regime. Significantly higher activities of SOD, CAT, POX, AsA and TPC with ‘0.2% Zn’ compared to foliar spray of other nutrients were quantified under all temperature regimes for most of cases over the years. However, ‘0.2% Zn’ mediated improvements in biosynthesis of SOD compared to water spray were 32% under sub optimal and 49% under supra optimal temperature regimes averaged across both years of study. Likewise, CAT, POX, AsA and TPC contents were improved by foliar spray of zinc under sub and supra optimal thermal regimes over water treated plant (Tables 5 and 6, Fig. 4). Statistically significant decrease in MDA contents were observed with ‘0.2% Zn’ and ‘1.5% K’ compared to other exogenous treatments under varying temperature regimes. However, remarkable change in biosynthesis of MDA was recorded with ‘0.2% Zn’ compared to other sprays. Foliar spray of ‘0.2% Zn’ instigated downregulation in MDA contents compared to water spray were 52% under sub optimal and 59% under supra optimal temperature regimes averaged across both years of study (Table 6, Fig. 4).

Chlorophyll *a*, *b* contents were reduced by 66%, 51% and 16%, 13%, respectively in the controls of supra and sub-optimal thermal regimes than the controls of optimal thermal regime averaged across of both years and of three development stages. Statistically alike and significantly more chlorophyll *a* and *b* biosynthesis was quantified with ‘0.2% Zn’ and ‘1.5% K’ compared to other foliar sprays under supra optimal temperature regime (Table 6, Fig. 5).

The Pn was reduced by 47% and 13% under the controls of supra and sub-optimal thermal regimes than the control of optimal thermal regime averaged across during both years of study and of three reproductive stages. Significantly more net photosynthetic rate, stomatal conductance, water potential and less osmotic potential were recorded with foliar ‘0.2% Zn’ and ‘1.5% K’ under supra optimal temperature regime over two years of experimentation (Table 7, Fig. 5).

The three nutrients (K, Zn and B averaged across) increased SCY under April and May thermal regimes by 15% and 17% than water spray averaged across during both years of study (Table 8, Fig. 6).

### Regression and correlation of studied components under both glasshouse and field conditions during 2012 and 2013

**Figure 7:**
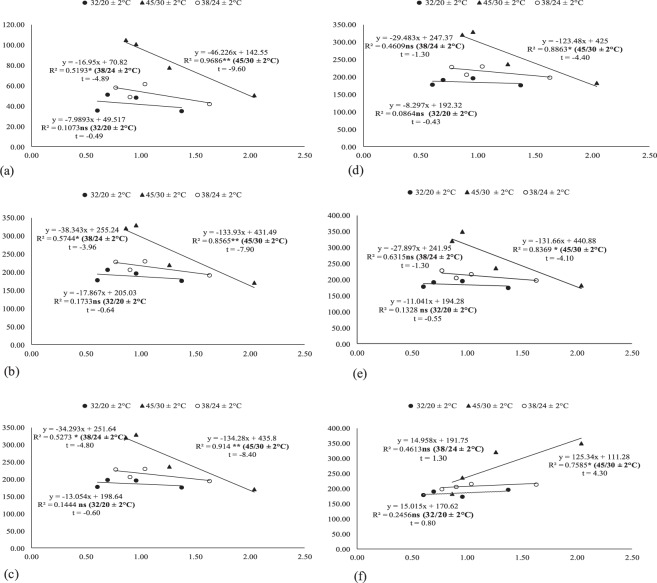
Association between malondialdehyde with (**a**) peroxidase, (**b**) ascorbic acid and (**c**) total phenolic contents, (**d**) stomatal conductance, (**e**) boll weight and (**f**) of stomatal conductance with water potential under glass house conditions (averaged across of three reproductive stages). * and ** indicates significance at 5 and 1% levels, respectively.

**Figure 8:**
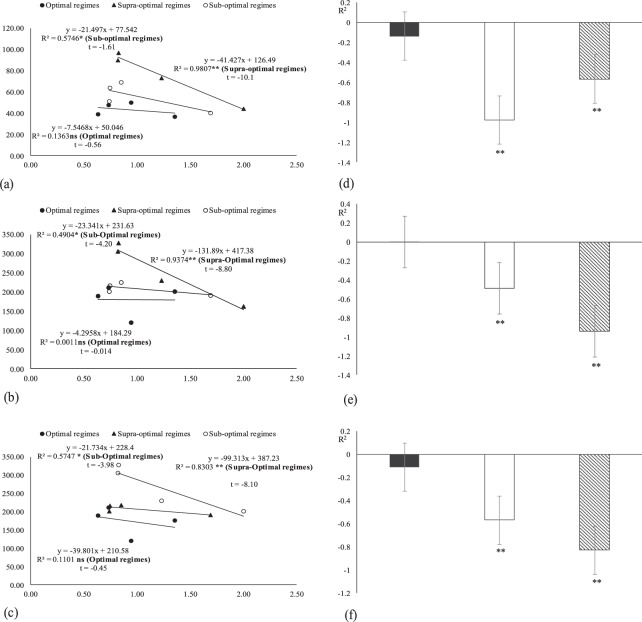
Association between malondialdehyde with (**a**) peroxidase, (**b**) ascorbic acid and (**c**) total phenolic contents under field conditions (averaged across of three reproductive stages). * and ** indicates significance at 5 and 1% levels, respectively.

**Figure 9:**
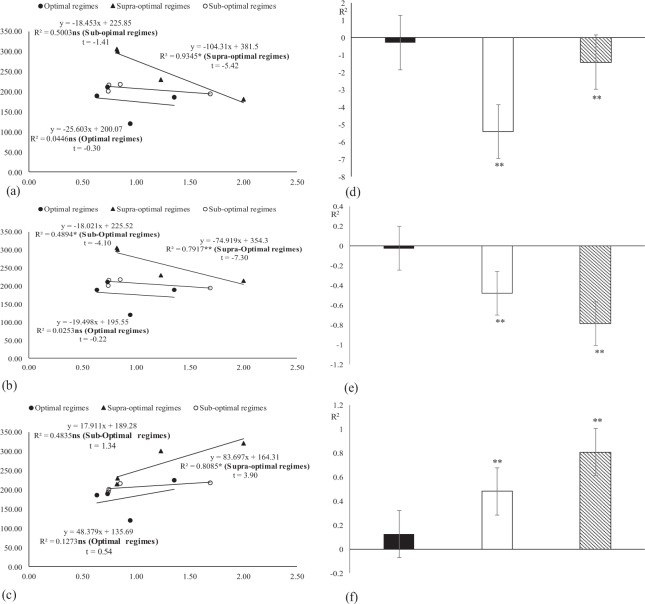
Association between malondialdehyde with (**a**) stomatal conductance, (**b**) boll weight and (**c**) of stomatal conductance with water potential under field conditions (averaged across of three reproductive stages). * and ** indicates significance at 5 and 1% levels, respectively.

The regression analysis under glasshouse conditions indicated that the malondialdehyde has strong negative relation with POX, AsA, TPC and with stomatal conductance. While water relations have positive relation with stomatal conductance (Fig. 7). The regression analysis under field conditions indicated that the malondialdehyde has strong negative relation with POX, AsA, TPC and with stomatal conductance while water relations have strong positive relation with stomatal conductance (Figs 8 and 9).

In glasshouse, Chlorophyll *a* and *b* have significant positive relationship with each other (*P* *<* *0.05* and *P* *<* *0.01*) and with CAT, SOD, Pn and SCY (*P* *<* *0.05* and *P* *<* *0.01*). Similarly, Pn and SCY have significantly positive relation with CAT, SOD and with chlorophyll *a* and *b* contents (Table 9). While in filed conditions, Chlorophyll *a* and *b* have significant positive relationship with each other (*P* *<* *0.05* and *P* *<* *0.01*) and with CAT, SOD, Pn and SCY (*P* *<* *0.05* and *P* *<* *0.01*). Similarly, Pn and SCY have significantly positive relation with CAT, SOD and with chlorophyll a, b contents (Table 10).

**Table 9:** Correlation between Chlor.a/b, Pn, CAT, SOD and SCY under glass house conditions.

Correlation components	CAT	Chlor a	Chlor b	Pn	SCY
Chlor a	0.47*				
Chlor b	0.48*	0.96**			
Pn	0.51*	0.98**	0.98**		
SCY	0.89**	0.71**	0.69**	0.68**	
SOD	0.76**	0.85**	0.82**	0.85**	0.67**

Chlor a (Chlorophyll a), Chlor b (Chlorophyll b), CAT (Catalase), SOD (Superoxide dismutase), Pn (Net photosynthetic rate) and SCY (Seed cotton yield).

*Correlation is significant at 0.01 levels.

**Correlation is significant at 0.05 level.

n (number of pairs of observations) = 48.

**Table 10:** Correlation between Chlor.a/b, Pn, CAT, SOD and SCY under field conditions during 2012 and 2013.

Parameters	Years	CAT	Chl a	Chl b	Pn	SCY
Chl a	2012	0.74**				
	2013	0.89**				
Chl b	2012	0.67**	0.94**			
	2013	0.77**	0.75**			
Pn	2012	0.85**	0.90**	0.63**		
	2013	0.72**	0.86**	0.68**		
SCY	2012	0.69**	0.70**	0.66**	0.80**	
	2013	0.67**	0.74**	0.59*	0.89**	
SOD	2012	0.87**	0.65**	0.71**	0.52*	0.63**
	2013	0.89**	0.65**	0.84**	0.79*8	0.69**

Chlor a (Chlorophyll a), Chlor b (Chlorophyll b), CAT (Catalase), SOD (Superoxide dismutase), Pn (Net photosynthetic rate) and SCY (Seed cotton yield).

*Correlation is significant at 0.01 levels.

**Correlation is significant at 0.05 levels.

n (number of pairs of observations) = 36.

Regression and correlation of studied components under glass house conditions.

## Discussion

Medium high to high temperature regimes influenced cotton crop physiology and the yield^14^. Reactive oxygen species (ROS) affected the membranes of each organelle for example the integrity of chloroplast/photosynthetic machinery^36,37^. A balance is required for ROS and antioxidants for the normal functions of plant defensive system. The oxidative stress causes the blockage of nutrients channels^5,38^. The high temperature regimes (45/30 °C) of glass house and (April and May) of field study upregulated the superoxide dismutase, catalase, peroxidase, ascorbic acid and total phenolic contents. However, cotton plants were unable to protect the cells from MDA contents due to oxidative stress^6^ indicating that the optimum temperature for biochemical and metabolic functions ranges between 25 to 31 °C^39^. In this study, the high temperature stress reduced chlorophyll contents, net photosynthetic rate and stomatal conductance^40,41^ indicating that photosynthetic apparatus is most sensitive to heat stress^42^. It may be the outcome of breakdown of photosynthetic pigments, associated proteins^43^, reduction in membrane integrity^44^, inefficiency of PS-II due to disruption of thylakoid structure^45^ and of linked enzymes^46^. The reduction in stomatal conductance under high temperature stress^47^ might be due to the stress signals from roots and consequent production of ethylene^48^ (Tables 3 and 7, Figs 2 and 5). In this study, leaf water potential was decreased while osmotic potential was increased under high temperature stress, as reported by^11^. It might be due to roots inability to up take water and nutrients under high temperature^49,50^. High temperature regimes of glass house and high temperature regimes (April and May thermal regimes) of field study showed reduction in averaged boll weight and seed cotton yield per plant as reported by^14^. This may be due to the production of stress hormones^51,52^ which may decrease the production and translocation of photo assimilates for developing bolls^53,54^.

The foliar spray of K, Zn and Boron activated the plant defensive system and reduced the MDA contents from medium to high temperature regimes (Tables 2 and 6; Figs 1 and 5) as reported by^21,55,56^. This may be due to the reduction in oxidative stress and the stress hormones^24,57^.

In this study, foliar application of K, Zn and B improved the light harvesting pigments (chlorophyll *a* and *b* contents)^58–60^ (Tables 3 and 6, Figs 2 and 5). This may be due to the alleviation of adverse impacts of oxidative stress that may boost the chlorophyll fluorescence and the energy output of light reactions^61,62^.

In this study, both potassium and zinc enhanced net photosynthetic rate and stomatal conductance^63,64^ might be due to the role of these nutrients in CO_2_ assimilation and in photosynthetic process^65–67^.

Foliar spray of potassium, zinc and boron increased water potential and reduced solute potential^68–70^ as found in this study which might be due to less relative cell injury of membranes^21,71,72^.

In this study, foliar spray of potassium, and zinc upsurged the boll weight and seed cotton yield even in K and Zn enriched soil^73,74^. This may be the outcome of the higher production of carbohydrates^75^. Under glass house and field studies, the chlorophyll (a and b) contents, Pn, stomatal conductance and antioxidants showed positive correlation with each other and with SCY while MDA showed strong negative relation with these parameters as reported by^6^.

## Conclusion

High temperature stress at three reproductive stages of cotton crop caused yield reduction which was due to lower boll weight that is associated to less chlorophyll contents and impaired photosynthesis. Exogenous application of macro and micro nutrients (K-1.5%, Zn-0.2% and B-0.1%) ameliorated the high temperature impact on cotton crop. These nutrients especially K, Zn and followed by B up-regulated the antioxidant enzymes (SOD, POX, CAT, AsA, phenolics and MDA), improved chlorophyll contents, net photosynthetic rate, water relations and seed cotton yield.
